# (*E*)-13-(4-Amino­phen­yl)parthenolide

**DOI:** 10.1107/S1600536813028730

**Published:** 2013-10-26

**Authors:** Narsimha Reddy Penthala, Venumadhav Janganati, Sean Parkin, Kottayil I. Varughese, Peter A. Crooks

**Affiliations:** a Department of Pharmaceutical Sciences, College of Pharmacy, University of Arkansas for Medical Sciences, Little Rock, AR 72205, USA; b Department of Chemistry, University of Kentucky, Lexington, KY 40506, USA; c College of Medicine, Department of Physiology & Biophysics, University of Arkansas for Medical Sciences, Little Rock, AR 72205, USA

## Abstract

The title compound, C_21_H_25_NO_3_ [systematic name: (3a*S*,9a*R*,10a*R*,10b*S*,*E*)-3-[(*E*)-4-(4-amino­benzyl­idene)-6,9a-dimethyl-3a,4,5,8,9,9a,10a,10b-octa­hydro­oxireno[2′,3′:9,10]cyclo­deca­[1,2-*b*]furan-2(3*H*)-one] was obtained from the reaction of parthenolide [synonym: 4,5-ep­oxy­germacra-1(10),11(13)-dieno-12,6-lactone] with 4-iodo­aniline under Heck reaction conditions. It was identified as the *E*-isomer (conformation about the exocyclic methyl­idene C=C bond; the conformation about the C=C bond in the ten-membered ring is also *E*). The mol­ecule is built up from fused ten-, five- (lactone) and three-membered (epoxide) rings with a 4-amino­phenyl group as a substituent. The ten-membered ring displays an approximate chair–chair conformation, while the lactone ring has an envelope conformation with the C atom bonded to the ring O atom as the flap. The dihedral angle between the benzene ring of the 4-amino­phenyl moiety and the lactone ring mean plane is 23.50 (8)°. In the crystal, mol­ecules are linked *via* N—H⋯O hydrogen bonds, between the amine group and the lactone and epoxide ring O atoms, forming chains propagating along the *b*-axis direction. Adjacent chains are linked *via* C—H⋯O inter­actions, forming an undulating two-dimensional network lying parallel to the plane (001). The absolute structure of the mol­ecule in the crystal was confirmed by resonance scattering [Flack parameter = 0.03 (3)].

## Related literature

For the biological activity of parthenolide, see: Hall *et al.* (1979 ▶). For the biological activity of parthenolide derivatives similar to the title compound, see: Hanson *et al.* (1970 ▶); Hehner *et al.* (1998 ▶); Kupchan *et al.* (1971 ▶); Nasim *et al.* (2011 ▶); Neelakantan *et al.* (2009 ▶); Oka *et al.* (2007 ▶); Ralstin *et al.* (2006 ▶); Rodriguez *et al.* (1976 ▶); Sun *et al.* (2006 ▶). For the synthesis and crystal structures of similar mol­ecules, see: Han *et al.* (2009 ▶).
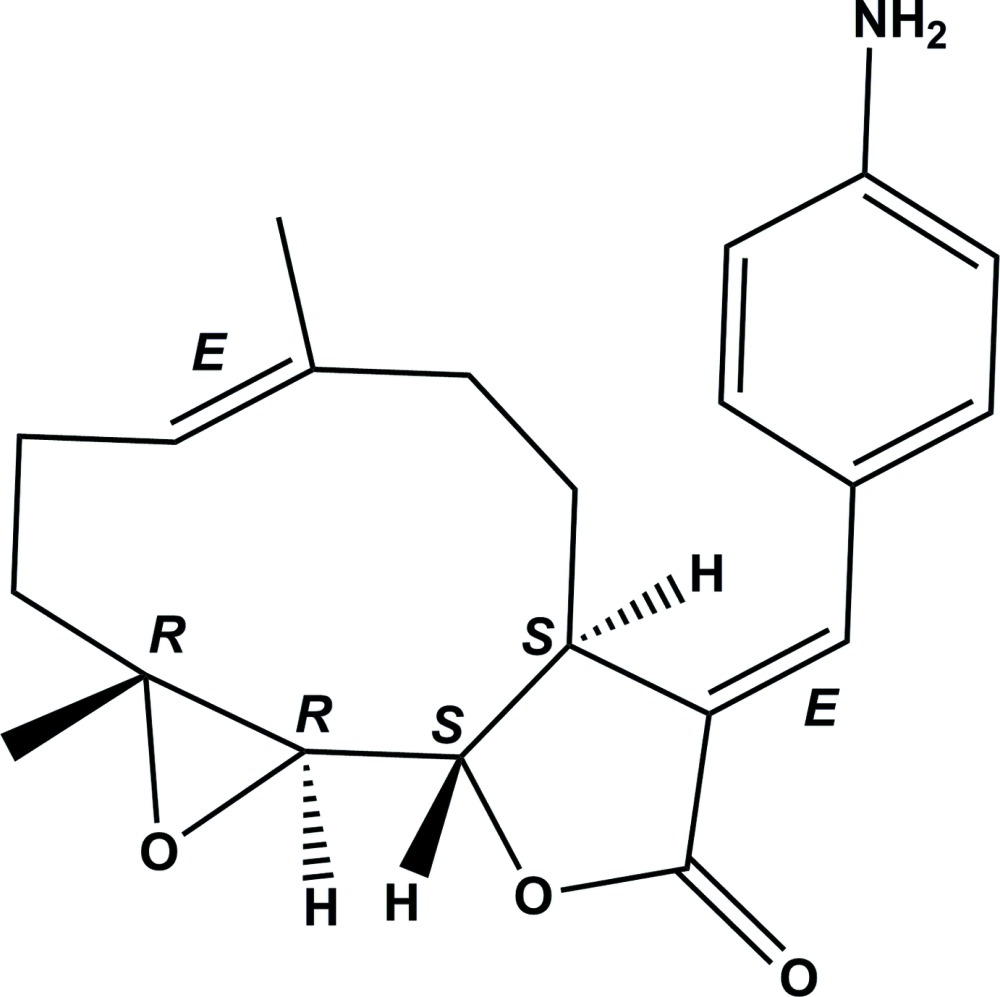


## Experimental

### 

#### Crystal data

C_21_H_25_NO_3_
*M* *_r_* = 339.42
Orthorhombic, 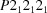

*a* = 8.5619 (1) Å
*b* = 11.8846 (2) Å
*c* = 17.7457 (3) Å
*V* = 1805.71 (5) Å^3^
*Z* = 4
Cu *K*α radiation
μ = 0.66 mm^−1^
*T* = 90 K
0.20 × 0.16 × 0.12 mm

#### Data collection

Bruker X8 Proteum diffractometer
Absorption correction: multi-scan (*SADABS*; Sheldrick, 1996 ▶) *T*_min_ = 0.843, *T*_max_ = 0.956
25400 measured reflections
3296 independent reflections
3267 reflections with *I* > 2σ(*I*)
*R*_int_ = 0.033

#### Refinement

*R*[*F* ^2^ > 2σ(*F* ^2^)] = 0.027
*wR*(*F* ^2^) = 0.070
*S* = 1.06
3296 reflections
234 parameters
H atoms treated by a mixture of independent and constrained refinement
Δρ_max_ = 0.13 e Å^−3^
Δρ_min_ = −0.14 e Å^−3^
Absolute structure: Flack *x* parameter determined using 1383 quotients [(*I* ^+^)−(*I* ^−^)]/[(*I* ^+^)+(*I* ^−^)] (Parsons *et al.*, 2013 ▶)
Absolute structure parameter: 0.03 (3)

### 

Data collection: *APEX2* (Bruker, 2006 ▶); cell refinement: *SAINT* (Bruker, 2006 ▶); data reduction: *SAINT*; program(s) used to solve structure: *SHELXS97* (Sheldrick, 2008 ▶); program(s) used to refine structure: *SHELXL2013* (Sheldrick, 2008 ▶); molecular graphics: *SHELXTL* (Sheldrick, 2008 ▶); software used to prepare material for publication: *SHELXTL*.

## Supplementary Material

Crystal structure: contains datablock(s) global, I. DOI: 10.1107/S1600536813028730/su2647sup1.cif

Structure factors: contains datablock(s) I. DOI: 10.1107/S1600536813028730/su2647Isup2.hkl

Additional supplementary materials:  crystallographic information; 3D view; checkCIF report

## Figures and Tables

**Table 1:** Hydrogen-bond geometry (Å, °)

*D*—H⋯*A*	*D*—H	H⋯*A*	*D*⋯*A*	*D*—H⋯*A*
N1—H1*A*⋯O1^i^	0.94 (2)	2.25 (3)	3.133 (2)	156 (2)
N1—H2*B*⋯O2^i^	0.90 (2)	2.57 (2)	3.077 (2)	116 (2)
C2—H2*A*⋯O3^ii^	0.99	2.63	3.372 (2)	132

Symmetry codes: (i) 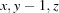; (ii) 
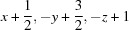
.

**Fig. 1.**
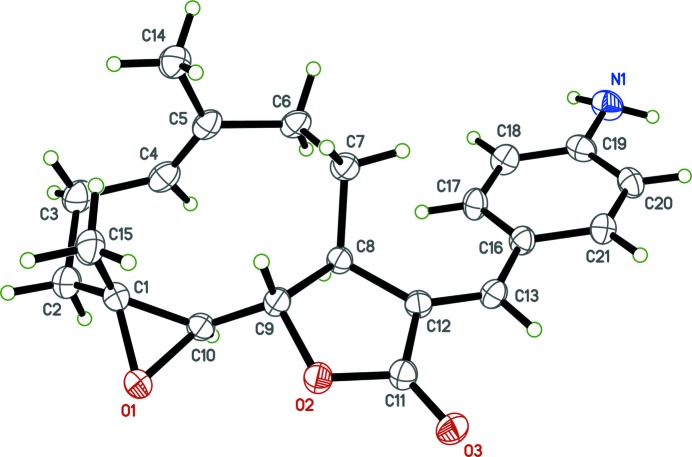
The molecular structure of the title molecule, with atom labelling. Displacement ellipsoids are drawn at the 50% probability level.

**Fig. 2.**
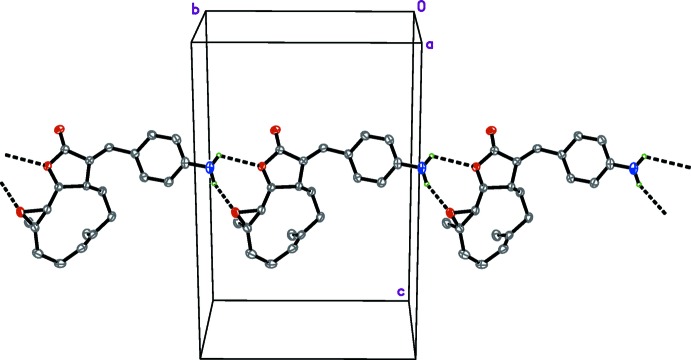
A partial crystal packing plot of the title compound showing the N-H···O hydrogen bonds that join the molecules into chains parallel to the b axis (see Table 1 for details; H atoms not involved in these hydrogen-bonds have been omitted to enhance clarity).

## supplementary crystallographic information

### 1. Comment

In recent years the parthenolide molecule [systematic name: (1aR,7aS,10aS,10bS,Z)-1a,5-dimethyl-8-methylene- 2,3,6,7,7a,8,10a,10b-octahydrooxireno[2',3':9,10]cyclodeca [1,2-b]furan-9(1aH)-one] and several structurally related sesquiterpene lactone analogues have been extensively studied due to their potent anti-tumor and cytotoxic properties (Oka *et al.*, 2007; Ralstin *et al.*, 2006; Sun *et al.*, 2006). The presence of an exo-methylene-γ-lactone functionality in these molecules is an important structural feature because of its exceptional reactivity toward nucleophilic functional groups (Rodriguez *et al.*, 1976). The exo-methylene-γ-lactone moiety was reported to be essential for significant cytotoxic activity (Kupchan *et al.*, 1971), for inhibition of nuclear factor κ-light-chain-enhancer of activated B cells (NF-κB; Hehner *et al.*, 1998) and for the antitumor activity of these sesquiterpene lactones (Hanson *et al.*, 1970).

Parthenolide is a sesquiterpene lactone of the germacranolide class which occurs naturally in the plant feverfew (*Tanacetum parthenium*), and is believed to be the active chemical constituent responsible for the plant's biological activity (Hall *et al.*, 1979). It has become a key intermediate for the synthesis of several novel antileukemic compounds over the past decade. From our research on antileukemia analogues of parthenolide, we have improved the poor water solubility properties of parthenolide and related sesquiterpenes by incorporating amino moieties at the exocyclic methylidene carbon *via* Michael addition (Neelakantan *et al.*, 2009). Also, in a recent communication we have reported the synthesis and antileukemic activity of a melampolide sesquiterpene lactone, melampomagnolide B (Nasim *et al.*, 2011).

The current study focuses on the synthesis of the title *E*-olefinic analogue of parthenolide which was obtained from the reaction of parthenolide with 4-iodoaniline utilizing Heck chemistry (Han *et al.*, 2009). In order to obtain detailed information on the structural conformation of the title compound and to establish the geometry of the exocyclic double bond, a single-crystal X-ray structure determination has been carried out.

The title molecule, Fig. 1, contains an *E*-exocyclic olefinic bond C12═C13. The ten-membered ring displays an approximate chair-chair conformation, while the lactone ring has an envelope conformation with atom C9 as the flap. The dihedral angle between the benzene ring of the 4-aminophenyl moiety and the lactone ring mean plane is 23.50 (8) °.

The crystal structures of the structurally related 3-trifluoromethylphenyl and 2-trifluoromethylphenyl derivatives of parthenolide have been reported previously (Han *et al.*, 2009). These molecules also have an *E*-conformation about the exocyclic olefinic double bond, and their absolute stereochemistries were also determined by resonance scattering. The lactone rings of these compounds have envelope conformations with the C-atom bonded to the O-atom as the flap, as in the title compound. The dihedral angles between the lactone ring mean plane and the aromatic rings of the 3-trifluoromethylphenyl and 2-trifluoromethylphenyl moieties are 40.52 (12) and 48.07 (12) °, respectively, compared to 23.50 (8) ° in the title compound.

Significant deviations from the ideal bond angle geometry around the carbon atoms, C12, C13 and C11, that are in the sp^2^ state and involved in the double bonds are observed; the C12═C13–C16, C13═C12–C8 and O3═ C11–C12 bond angles with the respective values of 131.43 (14), 132.77 (14), and 129.80 (14)°, deviate from the ideal value of 120°.

In the crystal, molecules are linked via N—H···O hydrogen bonds (Fig. 2 and Table 1), between the amine group and the lactone and epoxide ring O atoms, forming chains propagating along the *b*-axis. Adjacent chains are linked *via* C—H···O interactions (Table 1) forming undulating two-dimensional networks lying parallel to the plane (001).

### 2. Experimental

A mixture of parthenolide [Chemtek, Worcester, MA, USA](50 mg, 0.20 mmol), triethylamine (60 mg, 0.61 mmol), and 4-iodoaniline (48.56 mg, 0.22 mmol) in dimethylformamide (0.1 ml) was treated with palladium(II) acetate (0.5 mg, 0.002 mmol) and then stirred at 353 K for 24 h. Han *et al.* (2009) reported the synthesis of similar chiral molecules. The reaction mixture was cooled to room temperature, water (8 ml) was added, and the mixture was extracted with ethyl acetate (10 ml × 3). The separated organics were dried over Na_2_SO_4_ and concentrated under reduced pressure. The obtained crude residue was purified using silica flash chromatography (7:3, hexanes/EtOAc) to afford the title compound, which was recrystallized from a mixture of hexanes and ethyl acetate(1:1) as yellow needle-like crystals (40 mg, 58 % yield; M.p. = 512–514 K). Spectroscopic data for the title compound are available in the archived CIF.

### 3. Refinement

All the H-atoms were located in difference electron density maps. The NH_2_ H atoms were refined with *U*_iso_(H) = 1.5U_eq_(N). The C-bound H atoms were placed in idealized positions and refined as riding atoms: C-H = 0.98, 0.99, 1.00 and 0.95 Å for CH_3_, CH_2_, CH and C_sp_^2^H H atoms, respectively, with *U*_iso_(H) = 1.5*U*_eq_(C-methyl) and = 1.2U_eq_(C) for other H atoms.

### Crystal data

**Table:**

C_21_H_25_NO_3_	*D*_x_ = 1.249 Mg m^−^^3^
*M**_r_* = 339.42	Melting point = 512–514 K
Orthorhombic, *P*2_1_2_1_2_1_	Cu *K*α radiation, λ = 1.54178 Å
Hall symbol: P 2ac 2ab	Cell parameters from 9832 reflections
*a* = 8.5619 (1) Å	θ = 5.2–68.0°
*b* = 11.8846 (2) Å	µ = 0.66 mm^−^^1^
*c* = 17.7457 (3) Å	*T* = 90 K
*V* = 1805.71 (5) Å^3^	Block, colourless
*Z* = 4	0.20 × 0.16 × 0.12 mm
*F*(000) = 728	

### Data collection

**Table:**

Bruker X8 Proteum diffractometer	3296 independent reflections
Radiation source: fine-focus rotating anode	3267 reflections with *I* > 2σ(*I*)
Detector resolution: 5.6 pixels mm^-1^	*R*_int_ = 0.033
φ and ω scans	θ_max_ = 68.2°, θ_min_ = 5.7°
Absorption correction: multi-scan (*SADABS*; Sheldrick, 1996)	*h* = −10→10
*T*_min_ = 0.843, *T*_max_ = 0.956	*k* = −11→14
25400 measured reflections	*l* = −21→15

### Refinement

**Table:**

Refinement on *F*^2^	Secondary atom site location: difference Fourier map
Least-squares matrix: full	Hydrogen site location: difference Fourier map
*R*[*F*^2^ > 2σ(*F*^2^)] = 0.027	H atoms treated by a mixture of independent and constrained refinement
*wR*(*F*^2^) = 0.070	*w* = 1/[σ^2^(*F*_o_^2^) + (0.0401*P*)^2^ + 0.303*P*] where *P* = (*F*_o_^2^ + 2*F*_c_^2^)/3
*S* = 1.06	(Δ/σ)_max_ < 0.001
3296 reflections	Δρ_max_ = 0.13 e Å^−^^3^
234 parameters	Δρ_min_ = −0.14 e Å^−^^3^
0 restraints	Absolute structure: Flack parameter determined using 1383 quotients [(*I*^+^)-(*I*^-^)]/[(*I*^+^)+(*I*^-^)] (Parsons *et al.*, 2013)
Primary atom site location: structure-invariant direct methods	Absolute structure parameter: 0.03 (3)

### Special details

**Table:**

Experimental. Spectroscopic data for the title compound: ^1^H NMR (400 MHz, CDCl_3_): δ 7.54-7.55 (d, *J*=2.8 Hz, 1H), 7.23-7.26 (d, *J*=8.4 Hz, 2H), 6.68-6.70 (d, *J*=8.0 Hz, 2H), 5.28-5.30 (d, *J*=11.2 Hz, 1H), 4.02 (brs, 2H), 3.91-3.95 (t, *J*=8.0Hz, 1H), 3.25 (m, 1H), 2.82-2.84 (d, *J*=8.8 Hz, 1H), 2.28-2.45 (m, 1H), 2.14-2.24 (m, 5H), 1.69 (s, 3H), 1.61 (s, 1H), 1.43-1.45 (m, 1H), 1.31(s, 3H); ^13^C NMR (100 MHz,CDCl_3_) δ 171.63, 148.15, 138.64, 134.79, 132.17, 125.12, 124.03, 123.29, 114.42, 82.71, 66.62, 61.58, 46.98, 41.88, 36.10, 29.33, 24.33, 17.49, 17.36 *ppm*.
Geometry. All esds (except the esd in the dihedral angle between two l.s. planes) are estimated using the full covariance matrix. The cell esds are taken into account individually in the estimation of esds in distances, angles and torsion angles; correlations between esds in cell parameters are only used when they are defined by crystal symmetry. An approximate (isotropic) treatment of cell esds is used for estimating esds involving l.s. planes.

### Fractional atomic coordinates and isotropic or equivalent isotropic displacement parameters (Å^2^)

**Table:**

	*x*	*y*	*z*	*U*_iso_*/*U*_eq_	
O1	0.69671 (14)	0.83851 (9)	0.57032 (6)	0.0269 (3)	
O2	0.57735 (14)	0.71820 (9)	0.43765 (6)	0.0247 (3)	
O3	0.57901 (15)	0.65318 (10)	0.31945 (6)	0.0276 (3)	
N1	0.48608 (19)	−0.03270 (12)	0.45455 (10)	0.0323 (3)	
H1A	0.539 (3)	−0.055 (2)	0.4989 (14)	0.049*	
H2B	0.495 (3)	−0.077 (2)	0.4135 (14)	0.049*	
C1	0.61245 (19)	0.79003 (14)	0.63430 (9)	0.0255 (4)	
C2	0.7040 (2)	0.76874 (16)	0.70559 (9)	0.0321 (4)	
H1	0.7143	0.8397	0.7344	0.038*	
H2A	0.8102	0.7420	0.6926	0.038*	
C3	0.6202 (2)	0.67944 (15)	0.75440 (9)	0.0325 (4)	
H3A	0.6881	0.6579	0.7972	0.039*	
H3B	0.5227	0.7117	0.7752	0.039*	
C4	0.5821 (2)	0.57669 (14)	0.70835 (9)	0.0277 (4)	
H4	0.6688	0.5325	0.6928	0.033*	
C5	0.4420 (2)	0.53993 (13)	0.68657 (8)	0.0248 (3)	
C6	0.4284 (2)	0.44729 (13)	0.62863 (8)	0.0259 (3)	
H6A	0.3432	0.3957	0.6438	0.031*	
H6B	0.5268	0.4035	0.6282	0.031*	
C7	0.39551 (19)	0.49041 (13)	0.54839 (8)	0.0237 (3)	
H7A	0.3090	0.5455	0.5508	0.028*	
H7B	0.3597	0.4263	0.5172	0.028*	
C8	0.53600 (18)	0.54638 (12)	0.50866 (8)	0.0198 (3)	
H8	0.6346	0.5178	0.5321	0.024*	
C9	0.53593 (18)	0.67758 (12)	0.51252 (8)	0.0209 (3)	
H9	0.4293	0.7050	0.5263	0.025*	
C10	0.65311 (18)	0.72056 (12)	0.56849 (8)	0.0219 (3)	
H10	0.7423	0.6679	0.5775	0.026*	
C11	0.56586 (19)	0.63346 (13)	0.38588 (8)	0.0225 (3)	
C12	0.54277 (18)	0.52539 (12)	0.42479 (8)	0.0207 (3)	
C13	0.53493 (19)	0.43138 (13)	0.38311 (8)	0.0223 (3)	
H13	0.5363	0.4441	0.3303	0.027*	
C14	0.2884 (2)	0.58698 (15)	0.71301 (10)	0.0321 (4)	
H14A	0.3071	0.6429	0.7526	0.048*	
H14B	0.2236	0.5260	0.7331	0.048*	
H14C	0.2345	0.6227	0.6706	0.048*	
C15	0.4537 (2)	0.84179 (14)	0.64419 (9)	0.0301 (4)	
H15A	0.4639	0.9142	0.6702	0.045*	
H15B	0.3878	0.7913	0.6741	0.045*	
H15C	0.4057	0.8537	0.5947	0.045*	
C16	0.52461 (18)	0.31328 (13)	0.40450 (9)	0.0228 (3)	
C17	0.5641 (2)	0.27104 (12)	0.47583 (8)	0.0253 (3)	
H17	0.6003	0.3214	0.5136	0.030*	
C18	0.5517 (2)	0.15768 (13)	0.49238 (9)	0.0276 (3)	
H18	0.5777	0.1314	0.5414	0.033*	
C19	0.50076 (19)	0.08115 (13)	0.43735 (10)	0.0266 (3)	
C20	0.46429 (19)	0.12208 (14)	0.36578 (9)	0.0266 (3)	
H20	0.4306	0.0715	0.3276	0.032*	
C21	0.47670 (19)	0.23526 (13)	0.35002 (9)	0.0245 (3)	
H21	0.4521	0.2611	0.3008	0.029*	

### Atomic displacement parameters (Å^2^)

**Table:**

	*U*^11^	*U*^22^	*U*^33^	*U*^12^	*U*^13^	*U*^23^
O1	0.0341 (6)	0.0208 (5)	0.0258 (6)	−0.0064 (5)	0.0045 (5)	−0.0028 (5)
O2	0.0368 (6)	0.0174 (5)	0.0199 (5)	−0.0020 (5)	0.0000 (5)	0.0017 (4)
O3	0.0358 (6)	0.0279 (6)	0.0190 (5)	−0.0027 (5)	−0.0006 (5)	0.0043 (4)
N1	0.0364 (8)	0.0191 (7)	0.0415 (8)	−0.0011 (6)	0.0020 (7)	0.0013 (6)
C1	0.0310 (8)	0.0222 (7)	0.0232 (8)	−0.0057 (6)	0.0029 (6)	−0.0029 (6)
C2	0.0339 (9)	0.0375 (9)	0.0248 (8)	−0.0065 (8)	−0.0004 (7)	−0.0055 (7)
C3	0.0343 (9)	0.0430 (10)	0.0201 (8)	−0.0023 (8)	−0.0030 (7)	0.0005 (7)
C4	0.0320 (8)	0.0297 (8)	0.0212 (7)	0.0066 (7)	0.0028 (7)	0.0048 (6)
C5	0.0342 (9)	0.0220 (7)	0.0183 (7)	0.0041 (7)	0.0033 (7)	0.0048 (6)
C6	0.0346 (9)	0.0194 (7)	0.0236 (7)	0.0020 (7)	0.0069 (7)	0.0046 (6)
C7	0.0269 (8)	0.0203 (7)	0.0238 (8)	−0.0003 (6)	0.0024 (6)	0.0014 (6)
C8	0.0237 (8)	0.0164 (7)	0.0191 (7)	0.0006 (6)	0.0002 (6)	0.0006 (5)
C9	0.0266 (8)	0.0175 (7)	0.0185 (7)	0.0021 (6)	0.0016 (6)	0.0008 (5)
C10	0.0261 (7)	0.0177 (7)	0.0220 (7)	−0.0004 (6)	0.0016 (6)	0.0014 (6)
C11	0.0256 (8)	0.0206 (7)	0.0212 (7)	0.0012 (6)	−0.0012 (6)	−0.0002 (6)
C12	0.0229 (7)	0.0194 (7)	0.0197 (7)	−0.0002 (6)	−0.0007 (6)	0.0014 (6)
C13	0.0254 (8)	0.0233 (8)	0.0183 (7)	0.0023 (6)	−0.0002 (6)	0.0009 (6)
C14	0.0346 (9)	0.0279 (8)	0.0340 (9)	0.0007 (8)	0.0070 (7)	−0.0037 (7)
C15	0.0374 (9)	0.0242 (8)	0.0287 (8)	0.0011 (7)	0.0045 (7)	−0.0054 (7)
C16	0.0260 (8)	0.0196 (7)	0.0229 (7)	0.0015 (6)	0.0009 (6)	−0.0024 (6)
C17	0.0325 (8)	0.0198 (7)	0.0235 (7)	0.0036 (7)	−0.0022 (7)	−0.0033 (6)
C18	0.0329 (9)	0.0235 (7)	0.0265 (8)	0.0046 (7)	−0.0001 (7)	0.0014 (6)
C19	0.0242 (7)	0.0192 (7)	0.0365 (9)	0.0005 (6)	0.0031 (7)	−0.0004 (7)
C20	0.0256 (8)	0.0229 (8)	0.0314 (8)	−0.0004 (6)	−0.0019 (7)	−0.0060 (6)
C21	0.0265 (8)	0.0242 (8)	0.0229 (7)	0.0016 (6)	−0.0011 (6)	−0.0031 (6)

### Geometric parameters (Å, º)

**Table:**

O1—C10	1.4510 (18)	C8—C12	1.5101 (19)
O1—C1	1.4634 (19)	C8—C9	1.561 (2)
O2—C11	1.3667 (18)	C8—H8	1.0000
O2—C9	1.4573 (17)	C9—C10	1.501 (2)
O3—C11	1.2072 (18)	C9—H9	1.0000
N1—C19	1.393 (2)	C10—H10	1.0000
N1—H1A	0.94 (2)	C11—C12	1.472 (2)
N1—H2B	0.90 (2)	C12—C13	1.342 (2)
C1—C10	1.472 (2)	C13—C16	1.457 (2)
C1—C15	1.502 (2)	C13—H13	0.9500
C1—C2	1.510 (2)	C14—H14A	0.9800
C2—C3	1.547 (2)	C14—H14B	0.9800
C2—H1	0.9900	C14—H14C	0.9800
C2—H2A	0.9900	C15—H15A	0.9800
C3—C4	1.505 (2)	C15—H15B	0.9800
C3—H3A	0.9900	C15—H15C	0.9800
C3—H3B	0.9900	C16—C21	1.401 (2)
C4—C5	1.334 (3)	C16—C17	1.403 (2)
C4—H4	0.9500	C17—C18	1.383 (2)
C5—C14	1.504 (2)	C17—H17	0.9500
C5—C6	1.511 (2)	C18—C19	1.404 (2)
C6—C7	1.539 (2)	C18—H18	0.9500
C6—H6A	0.9900	C19—C20	1.395 (2)
C6—H6B	0.9900	C20—C21	1.378 (2)
C7—C8	1.545 (2)	C20—H20	0.9500
C7—H7A	0.9900	C21—H21	0.9500
C7—H7B	0.9900		
			
C10—O1—C1	60.67 (10)	C10—C9—C8	111.63 (12)
C11—O2—C9	110.56 (11)	O2—C9—H9	109.7
C19—N1—H1A	114.0 (15)	C10—C9—H9	109.7
C19—N1—H2B	112.6 (15)	C8—C9—H9	109.7
H1A—N1—H2B	118 (2)	O1—C10—C1	60.08 (9)
O1—C1—C10	59.25 (9)	O1—C10—C9	121.02 (12)
O1—C1—C15	112.05 (13)	C1—C10—C9	123.89 (14)
C10—C1—C15	122.43 (14)	O1—C10—H10	113.8
O1—C1—C2	117.39 (14)	C1—C10—H10	113.8
C10—C1—C2	116.62 (15)	C9—C10—H10	113.8
C15—C1—C2	116.15 (14)	O3—C11—O2	120.44 (14)
C1—C2—C3	110.08 (14)	O3—C11—C12	129.80 (14)
C1—C2—H1	109.6	O2—C11—C12	109.72 (12)
C3—C2—H1	109.6	C13—C12—C11	118.35 (13)
C1—C2—H2A	109.6	C13—C12—C8	132.77 (14)
C3—C2—H2A	109.6	C11—C12—C8	108.87 (12)
H1—C2—H2A	108.2	C12—C13—C16	131.43 (14)
C4—C3—C2	110.68 (13)	C12—C13—H13	114.3
C4—C3—H3A	109.5	C16—C13—H13	114.3
C2—C3—H3A	109.5	C5—C14—H14A	109.5
C4—C3—H3B	109.5	C5—C14—H14B	109.5
C2—C3—H3B	109.5	H14A—C14—H14B	109.5
H3A—C3—H3B	108.1	C5—C14—H14C	109.5
C5—C4—C3	128.15 (15)	H14A—C14—H14C	109.5
C5—C4—H4	115.9	H14B—C14—H14C	109.5
C3—C4—H4	115.9	C1—C15—H15A	109.5
C4—C5—C14	125.04 (14)	C1—C15—H15B	109.5
C4—C5—C6	120.33 (15)	H15A—C15—H15B	109.5
C14—C5—C6	114.58 (15)	C1—C15—H15C	109.5
C5—C6—C7	113.64 (12)	H15A—C15—H15C	109.5
C5—C6—H6A	108.8	H15B—C15—H15C	109.5
C7—C6—H6A	108.8	C21—C16—C17	117.15 (14)
C5—C6—H6B	108.8	C21—C16—C13	118.40 (14)
C7—C6—H6B	108.8	C17—C16—C13	124.42 (14)
H6A—C6—H6B	107.7	C18—C17—C16	121.43 (15)
C6—C7—C8	115.03 (13)	C18—C17—H17	119.3
C6—C7—H7A	108.5	C16—C17—H17	119.3
C8—C7—H7A	108.5	C17—C18—C19	120.49 (15)
C6—C7—H7B	108.5	C17—C18—H18	119.8
C8—C7—H7B	108.5	C19—C18—H18	119.8
H7A—C7—H7B	107.5	N1—C19—C20	121.19 (15)
C12—C8—C7	114.12 (13)	N1—C19—C18	120.33 (16)
C12—C8—C9	102.02 (11)	C20—C19—C18	118.47 (15)
C7—C8—C9	114.19 (12)	C21—C20—C19	120.52 (15)
C12—C8—H8	108.7	C21—C20—H20	119.7
C7—C8—H8	108.7	C19—C20—H20	119.7
C9—C8—H8	108.7	C20—C21—C16	121.91 (15)
O2—C9—C10	109.13 (12)	C20—C21—H21	119.0
O2—C9—C8	106.91 (11)	C16—C21—H21	119.0
			
C10—O1—C1—C15	−115.61 (15)	C8—C9—C10—O1	166.95 (12)
C10—O1—C1—C2	106.19 (17)	O2—C9—C10—C1	121.71 (15)
O1—C1—C2—C3	−158.44 (14)	C8—C9—C10—C1	−120.33 (15)
C10—C1—C2—C3	−91.06 (18)	C9—O2—C11—O3	171.96 (15)
C15—C1—C2—C3	65.05 (19)	C9—O2—C11—C12	−9.99 (16)
C1—C2—C3—C4	51.03 (19)	O3—C11—C12—C13	0.4 (3)
C2—C3—C4—C5	−110.84 (19)	O2—C11—C12—C13	−177.46 (14)
C3—C4—C5—C14	−8.7 (3)	O3—C11—C12—C8	179.14 (17)
C3—C4—C5—C6	168.64 (15)	O2—C11—C12—C8	1.33 (18)
C4—C5—C6—C7	−98.81 (18)	C7—C8—C12—C13	−50.8 (2)
C14—C5—C6—C7	78.79 (18)	C9—C8—C12—C13	−174.49 (17)
C5—C6—C7—C8	73.72 (17)	C7—C8—C12—C11	130.63 (13)
C6—C7—C8—C12	144.91 (13)	C9—C8—C12—C11	6.96 (17)
C6—C7—C8—C9	−98.26 (15)	C11—C12—C13—C16	174.88 (16)
C11—O2—C9—C10	135.27 (13)	C8—C12—C13—C16	−3.6 (3)
C11—O2—C9—C8	14.39 (16)	C12—C13—C16—C21	163.27 (17)
C12—C8—C9—O2	−12.50 (15)	C12—C13—C16—C17	−18.8 (3)
C7—C8—C9—O2	−136.12 (12)	C21—C16—C17—C18	−2.0 (2)
C12—C8—C9—C10	−131.78 (13)	C13—C16—C17—C18	−179.96 (16)
C7—C8—C9—C10	104.60 (15)	C16—C17—C18—C19	0.9 (3)
C1—O1—C10—C9	113.87 (16)	C17—C18—C19—N1	−179.03 (16)
C15—C1—C10—O1	98.02 (16)	C17—C18—C19—C20	0.4 (2)
C2—C1—C10—O1	−107.50 (15)	N1—C19—C20—C21	178.82 (16)
O1—C1—C10—C9	−109.26 (15)	C18—C19—C20—C21	−0.6 (2)
C15—C1—C10—C9	−11.2 (2)	C19—C20—C21—C16	−0.5 (2)
C2—C1—C10—C9	143.25 (15)	C17—C16—C21—C20	1.8 (2)
O2—C9—C10—O1	49.00 (18)	C13—C16—C21—C20	179.88 (15)

### Hydrogen-bond geometry (Å, º)

**Table:**

*D*—H···*A*	*D*—H	H···*A*	*D*···*A*	*D*—H···*A*
N1—H1*A*···O1^i^	0.94 (2)	2.25 (3)	3.133 (2)	156 (2)
N1—H2*B*···O2^i^	0.90 (2)	2.57 (2)	3.077 (2)	116 (2)
C2—H2*A*···O3^ii^	0.99	2.63	3.372 (2)	132

Symmetry codes: (i) *x*, *y*−1, *z*; (ii) *x*+1/2, −*y*+3/2, −*z*+1.
